# Evaluating Nitrogen Reduction Under Combined Rice Straw Biochar and Milk Vetch Application: A Multi-Objective Assessment of Rice Yield, Grain Quality, and Partial Factor Productivity of Nitrogen Fertilizer in a Double-Rice Cropping System

**DOI:** 10.3390/foods15132354

**Published:** 2026-07-02

**Authors:** Zhijian Xie, Hanghang Wang, Kun Zhang, Ye Lu

**Affiliations:** 1 College of Land Resources and Environment, Jiangxi Agricultural University, Nanchang 330045, China; 19993827049@163.com (H.W.); 15288031876@163.com (Y.L.); 2 Jiangxi Institute of Red Soil and Germplasm Resources, Nanchang 331717, China; zhkp1984@163.com

**Keywords:** rice straw biochar, milk vetch, N reduction, rice yield and quality, Entropy-weighted TOPSIS

## Abstract

Co-incorporating rice straw biochar (RSB) and milk vetch (MV) is a promising strategy for sustainable rice production. However, the appropriate nitrogen (N) reduction rate under RSB + MV, particularly when balancing multiple production objectives, remains unclear. This study evaluated five N-reduction rates (0–70% conventional N fertilizer) under RSB + MV. Grain yield, quality, nutrient uptake, and partial factor productivity of N fertilizer (*PFP_N_*) were assessed in early and late rice. Entropy-weighted Technique for Order Preference by Similarity to Ideal Solution (EW-TOPSIS) model was used to integrate all indicators, when Gaussian model was applied to estimate the appropriate N-reduction rate. Pearson correlation and random forest analysis identified primary predictors of grain quality. Results showed that appropriate N-reduction (≤30%) under RSB + MV maintained or increased grain yield, quality, and *PFP_N_* compared to conventional N fertilization alone. Among all treatments, N_80_BM and N_70_BM achieved the highest comprehensive scores in EW-TOPSIS analysis, and Gaussian model-estimated N-reduction of 18.2% and 21.0% for early and late rice, respectively. Nutrient uptake, effective panicle number and filled grains per panicle were identified as primary predictors of grain quality. Therefore, under the tested condition, RSB + MV with 20% N-reduction achieved the highest overall performance. However, these findings require further validation across multiple-year and multi-location.

## 1. Introduction

With continued global population growth and shifts in dietary patterns, global food demand is projected to increase by 35–56% by 2050 compared with 2010 levels [1]. The application of organic amendments, such as biochar and milk vetch (MV), can significantly improve crop yield and nitrogen (N) use efficiency (NUE) [2]. Reddish paddy soils, a key component of the double-rice production system in southern China, play a crucial role in ensuring national food security. However, long-term intensive cultivation and improper N management have not sustainably increased rice yield. Moreover, these practices have led to low NUE, increased environmental pollution, and potential declines in rice quality, thereby hindering the sustainable development of rice production [3].

Rational N management is crucial for improving rice yield, grain quality, and economic returns [4,5]. High rice yields are typically accompanied by the production of large quantities of straw [6]. However, improper straw disposal practices, such as open burning or discarding, increase PM_2.5_ emissions and may exacerbate the incidence of diseases and insect pests in subsequent crops [7]. Although long-term straw incorporation can increase soil carbon (C) and N contents and sustain stable grain yields for up to 13 years, it may induce adverse effects such as soil acidification [8,9]. Biochar, produced through straw carbonization and subsequent soil application, provides an effective approach for overcoming the limitations associated with straw burning and direct straw return. Owing to its stable aromatic C structure, high porosity, and large specific surface area, biochar can improve soil physicochemical properties, enhance nutrient retention and availability, and regulate microbial activity. These benefits promote crop nutrient uptake, increase crop yield, and enhance crop quality [10,11]. Compared with long-term sole N fertilization, the combined application of biochar and N fertilizer can enhance C–N interactions and modify soil pore structure through the redistribution of organic C within soil aggregates. These changes promote root growth and improve crop yield [12,13]. Additionally, compared with direct straw return, straw carbonization is more effective in sustainably improving crop yield and NUE in rice-based cropping systems [14]. However, the increase in rice yield resulting from biochar application may be insufficient to offset its high production, storage, and transportation costs. Consequently, biochar applications may reduce the net economic benefits of paddy ecosystems [15].

Given these challenges, biological tillage based on the nutrient-supply and soil-improvement functions of green manure (GM, e.g., MV) provides an effective strategy for enhancing soil health, rice quality, crop yield, and production efficiency in paddy soils [16,17]. MV, a major source of N and organic matter in rice-growing regions of southern China, plays a vital role in improving soil fertility, reducing dependence on N fertilizers and greenhouse gas emissions, and increasing rice yield and NUE. Therefore, MV has emerged as a key strategy for enhancing the sustainability and productivity of double-cropping rice systems [18]. Studies have shown that crop N uptake depends on the synchronization between soil N availability and crop N demand [19]. The combined application of GM and N fertilizer improves soil nutrient availability and balance, enhances the synchronization of soil N supply with crop N demand, and optimizes the rhizosphere microenvironment. These effects promote rice N uptake and utilization, improve yield components, and increase rice yield and NUE [18]. Rice preferentially absorbs NH_4_^+^. Xia et al. (2025) reported that MV application enhances organic N cycling between soil and rice plants and significantly increases NH_4_^+^ content in paddy soil [20]. Consequently, soil N supply capacity is improved, thereby promoting more efficient utilization of native soil N and increasing rice yield.

The combined application of rice straw biochar (RSB) and MV in paddy fields creates a novel “C–N synergy” model. In this system, RSB provides favorable physicochemical “habitats” for soil microorganisms and enhances N retention. MV continuously supplies active N and labile C. These complementary functions improve nutrient availability, optimize plant N uptake and utilization, and increase rice yield [19,21].

Rice grain quality directly affects its utilization, market acceptance, and economic value. With continuous improvements in living standards, consumer demand has gradually shifted from high yield alone to both high yield and superior grain quality, leading to increased focus on rice quality [22,23,24]. Reducing N fertilizer inputs without compromising grain yield and quality remains a key challenge for sustainable rice production. The incorporation of organic amendments (e.g., biochar and GM) has emerged as a promising strategy for maintaining high yields and enhancing environmental sustainability [25,26]. However, the appropriate N-reduction rate and the underlying mechanisms of RSB + MV co-application, particularly regarding the balance among multiple production objectives (e.g., yield, quality, and NUE), remain unclear. Hence, this study hypothesized that a moderate reduction in N input under the combined application of RSB and MV could maintain grain yield, grain quality, and NUE. Conversely, N reduction beyond a critical threshold may significantly impair the overall performance of double-rice cropping systems. The findings from a single-site, fourth-year field experiment provide both methodological insights and practical guidance for optimizing N management under the integrated management practice of RSB + MV + reduced N in reddish paddy fields of southern China.

## 2. Materials and Methods

### 2.1. Experimental Site, and Soil

The field experiment was initiated in 2021 at Jiangxi Institute of Red Soil and Germplasm Resources in Jinxian, China (116°16′ E, 28°20′ N; Figure S1), which has a subtropical monsoon climate. In 2024, the mean annual temperature was 20.8 °C, the annual precipitation was 1819.1 mm, and the accumulated temperature above 10 °C was 5950.0 °C. The frost-free period was 262 days, and the annual sunshine duration was 1862.5 h.

According to the World Reference Base for Soil Resources (WRB, 2015), the experimental soil was classified as Anthrosols (Hydragric) and was derived from Quaternary red clay with a clay loam texture. The topsoil (0–20 cm) had a pH (soil/water = 1:2.5) of 5.23. The contents of soil organic carbon (SOC), total N (TN), alkaline hydrolysable N (AHN), available P (AP), and available K (AK) were 19.3 g/kg, 1.51 g/kg, 176.9 mg/kg, 31.6 mg/kg, and 75.7 mg/kg, respectively. A schematic of the field experimental layout is presented in Figure S2.

### 2.2. Rice Straw Biochar (RSB) and Milk Vetch (MV)

MV (*Astragalus sinicus* L. cv. Ganzi No.1) was annually sown in mid-to-late October at a rate of 30 kg/hm^2^ in all plots except the N_100_ plots. No additional fertilizers were applied during the MV growing season, regardless of whether the fields were left fallow or planted with MV.

Rice straw, collected immediately after the annual rice harvest, was used as feedstock for biochar production. The straw was pyrolyzed at 450–500 °C for 2 h at a heating rate of ∼20 °C/min under oxygen-limited conditions to produce RSB. Before application, RSB was passed through a 5 mm sieve.

The total C and N contents of MV and RSB were determined using an elemental analyzer (Vario Max, Elementar, Hanau, Germany). The pH of RSB was measured at a solid-to-liquid ratio of 1:2.5 (*w*/*v*). The specific surface area of RSB was determined using the Brunauer–Emmett–Teller method based on N_2_ sorption at 77 K with a specific surface area analyzer (TriStar II 3020, Micromeritics, Norcross, GA, USA). The physicochemical properties of MV and RSB are presented in Table 1.

### 2.3. Experimental Design and Field Management

This study was designed to evaluate the effects of N reduction under the combined RSB + MV system, rather than to quantify the individual contributions of RSB and MV. Our previous studies [21,27] have shown that under MV incorporation in paddy fields, applying 80% of the N fertilizer rate substantially increased rice yield. However, applying 60% N resulted in yield loss. Additionally, the combined application of RSB and MV with 80% N fertilizer increased both rice yield and NUE. According to these findings, we hypothesized that a 20–30% reduction in N output under the RSB + MV system would represent a “safe range” for maintaining rice yield. A 50% reduction in N output was considered a critical yield threshold. A 70% reduction in N output was included as an extreme treatment. Consequently, six experiments were established in a randomized design with three replicates: a conventional N fertilizer treatment (N_100_) and five RSB + MV treatments at 100%, 80%, 70%, 50%, and 30% of the conventional N fertilizer rate. These treatments were designated as N_100_BM, N_80_BM, N_70_BM, N_50_BM, and N_30_BM. Thus, N_100_ represented conventional chemical N fertilizer without RSB + MV, whereas N_100_BM to N_30_BM represented the combined RSB + MV package at decreasing chemical N rates. Each plot had an area of 20 m^2^ and was separated by a black plastic film-covered ridge. A 1 m buffer zone was maintained around each plot.

Twenty-five-day-old seedlings of early rice (*Oryza sativa* L. cv. Xiangzaoxian 45) were transplanted in late April at 15 cm × 30 cm spacing with four seedlings per hill and harvested in mid-July. Thirty-day-old seedlings of late rice (*Oryza sativa* L. cv. Taiyou 871) were transplanted in late July at 24 cm × 22 cm spacing with two seedlings per hill and harvested in late October. For both early and late rice, alternate wetting and drying and intermittent irrigation were adopted during the early and late growth stages, respectively.

The recommended fertilizer rates for early and late rice were 150 and 180 kg N/hm^2^ (urea, 46.4% N), 75 and 75 kg P_2_O_5_/hm^2^ (superphosphate, 12.0% P_2_O_5_), and 120 and 150 kg K_2_O/hm^2^ (potassium chloride, KCl, 60.0% K_2_O). MV was pulverized and incorporated into the 0–20 cm soil layer at 22.5 t/hm^2^ at the full-bloom stage, 5 days before early-rice transplanting. The field was then flooded to ~5 cm. RSB was applied at 4 t/hm^2^ on the same day as MV incorporation. No additional RSB or other organic amendments were applied during the late rice season, while chemical fertilizer rates remained consistent across treatments.

For both early and late rice, 50% of the urea was applied as basal fertilizer, 20% was top-dressed at the tillering stage, and the remaining 30% was top-dressed at the panicle initiation stage. P and K fertilizers were applied as basal fertilizers at the recommended rates. All basal fertilizers were incorporated into the paddy soil one day before rice transplanting.

### 2.4. Rice Grain Yield, Yield Components, and Nutrients Content Testing

Preliminary monitoring of topsoil (0–20 cm) properties during the first three years indicated that SOC, total nitrogen, and available nutrient contents approached a dynamic equilibrium by the third year. Treatment differences stabilized, and interannual variability was substantially reduced. Consequently, after a treatment-establishment period, the fourth year (2024) of the field experiment was selected for comprehensive measurements of rice yield, N uptake and utilization, and grain quality. Therefore, all data reported in this study were collected during the 2024 growing season. The results provide a high-resolution assessment of optimized N management under the RSB + MV system without the confounding effects of transitional soil changes.

In 2024, sixty rice hills were surveyed in each subplot to determine the average number of effective panicles. An additional five 5 hills were sampled from each subplot at maturity. During manual threshing, filled and unfilled grains were separated using an air separator. Spikelets per panicle, filled grain percentage, and 1000-grain weight were determined.

All plots were harvested at the physiological maturity of early and late rice in mid-July and mid-to-late October, respectively, for grain yield determination. Grain yield was adjusted to a moisture content of 13.5% and expressed as ×10^3^ kg/hm^2^. Subsequently, rice grain samples were collected to determine N, P, and K contents according to the method described by Xie et al. (2022) [21]. The N-use efficiency (NUE) was evaluated using the partial factor productivity of N fertilizer (*PFP_N_*, kg/kg), which was calculated according to Equation (1).

(1) $${PFP}_{N}(kg/kg)=\frac{Y}{F}$$

where *Y* is the gain yields (kg/hm^2^) of early rice or late rice, and *F* represents application rate of nitrogen fertilizer (kg/hm^2^) of early rice or late rice.

### 2.5. Rice Grain Quality and Rapid Visco-Analyzer (RVA) Profiles

To determine the average number of panicles, rice plants within a 4 m^2^ area were surveyed in each plot at physiological maturity. According to this assessment, six hills were sampled from each plot for subsequent analyses. During manual threshing, filled and unfilled grains were separated using an air separator. The number of filled grains per panicle and the 1000-grain weight were then calculated.

Sun-dried rice grains were stored for 3 months after harvest at ~13% moisture content. Before quality analysis, empty and shriveled grains were removed. Approximately 1 kg of rice was randomly sampled from each treatment in triplicate and processed using a dehuller and a laboratory rice mill.

Appearance quality (chalky grain rate, chalkiness degree, and length-to-width ratio of head-milled grain), milling quality (brown rice rate, milled rice rate, and head-milled rice rate), physicochemical quality (e.g., gel consistency, amylose content, and protein content), and cooked-rice eating quality value were determined according to the method described by Wang et al. (2025) [28]. RVA profile characteristics (peak viscosity, trough viscosity, breakdown viscosity, and setback viscosity) were determined according to the method described by Chen et al. (2023) [11].

### 2.6. Multi-Objective Evaluation Using the Entropy-Weighted Technique for Order Performance by Similarity to an Ideal Solution (EW-TOPSIS) Model

Entropy weighting (EW) determines indicator weights according to the degree of variation among indicators based on information entropy. The weights were calculated separately for early and late rice [29]. The TOPSIS method evaluates alternatives according to their distances from the positive- and negative-ideal solutions. The EW–TOPSIS model determines indicator weights, calculates the relative closeness of each treatment to the ideal solution, and ranks treatment performance. This method eliminates subjective weight assignments and provides objective, interpretable, and computationally flexible results [30]. A total of 21 indicators were separately selected for the multi-objective evaluation of early or late rice. These indictors included grain yield (positive), yield components (effective panicle number, filled grains per panicle, and 1000-grain weight; positive), nutrient accumulation (N, P, and K; positive), *PFP_N_* (positive), milling quality (brown rice rate, milled rice rate, and head rice rate; positive), appearance quality (chalky grain rate, negative; chalkiness degree, negative; length-to-width ratio of head rice; positive), cooking quality (gel consistency, positive; amylose content, negative; protein content, negative), and RVA profile traits (peak viscosity and breakdown viscosity, positive; hot viscosity and setback viscosity, negative). The full list of indicators, their directions, entropy weights, normalized values, and weighted normalized values for early rice and late rice are provided in Supplementary Tables S1–S5, respectively. No explicit variable filtering was performed before EW because indicators with low information variation are inherently assigned lower weights.

For r schemes and s evaluation indicators, the original data were expressed as the matrix $A={\left(A_{ij}\right)}_{n\times s}$. Here, $A_{ij}$ represents the value of the j indicator for the i scheme.

To eliminate the effects of differences in scale and units among indicators, all evaluation indicators were normalized using a max–min method. Positive and negative indicators were normalized using Equations (2) and (3), respectively, yielding the normalized matrix $B={\left(B_{ij}\right)}_{n\times s}$.

(2) $$B_{ij}=\frac{A_{ij}-{Min}_{A_{ij}}}{{Max}_{A_{ij}}-{Min}_{A_{ij}}}$$

(3) $$B_{ij}=\frac{{Max}_{A_{ij}}-A_{ij}}{{Max}_{A_{ij}}-{Min}_{A_{ij}}}$$

where i=1,2,……,n, and j=1,2,……,s. $B_{ij}$ represents the normalized value of the *j* indicator for the *i* scheme, and $A_{ij}$ denotes the original value.

The information entropy of the *j* indicator for the *i* scheme $\left(E_{ij}\right)$ was calculated as follows. First, the proportion of each normalized value under the same indicator was calculated using Equation (4).

(4) $$P_{ij}=\frac{B_{ij}}{\sum_{i=1}^{n}B_{ij}}$$

Second, the information entropy for each indicator was determined by Equation (5).

(5) $$E_{j}=-\frac{\sum_{i=1}^{n}\left(P_{ij}\times lnP_{ij}\right)}{lnn}$$

where $E_{j}$ was the information entropy value of the *j* indicator, and *n* is the number of evaluation schemes.

Then, the weight of each indicator was calculated by Equation (6).

(6) $$W_{j}=\frac{1-E_{j}}{\sum_{i=1}^{n}\left(1-E_{j}\right)}$$

where $W_{j}$ was the weight coefficient of the *j* indicator.

Subsequently, the weighted normalized matrix was constructed using Equation (7).

(7) $$R_{ij}=W_{j}\times P_{ij}$$

The positive ideal solution $\left(R_{j}^{+}\right)$ and the negative-ideal solution $\left(R_{j}^{-}\right)$ were determined using Equations (8) and (9), respectively.

(8) $$R_{j}^{+}=max(R_{11},R_{12},R_{13},\cdots \cdots ,R_{1n})$$

(9) $$R_{j}^{-}=min(R_{11},R_{12},R_{13},\cdots \cdots ,R_{1n})$$

The Euclidean distances (ED) between each scheme and the positive and negative-ideal solutions were calculated using Equations (10) and (11), respectively.

(10) $${ED}_{i}^{+}=\sqrt{\sum_{j=1}^{n}{\left(R_{ij}-R_{j}^{+}\right)}^{2}}$$

(11) $${ED}_{i}^{-}=\sqrt{\sum_{j=1}^{n}{\left(R_{ij}-R_{j}^{-}\right)}^{2}}$$

where ${ED}_{i}^{+}$ and ${ED}_{i}^{-}$ were the Euclidean distances to the positive and negative-ideal solutions, respectively, and i=1,2,……,n.

The relative closeness $\left(RC_{i}\right)$ of the i scheme to the positive ideal solution was calculated using Equation (12). The schemes were ranked according to their ${RC}_{i}$ values.

(12) $${RC}_{i}=\frac{{ED}_{i}^{-}}{{ED}_{i}^{+}+{ED}_{i}^{-}}$$

The ${RC}_{i}$ ranges from 0 to 1. Larger ${RC}_{i}$ values indicate that the corresponding scheme is closer to the ideal solution [31].

A Gaussian function was used to model the relationship between ${RC}_{i}$ (*Y*) and application rate of N fertilizer (*X*) based on the experimental data. The model was developed separately for early rice and late rice to estimate the appropriate application rate of N fertilizer under the RSB + MV system for the multi-objective optimization of grain yield, grain quality, and NUE. Despite the high R^2^ values, the estimated optimum requires further validation owing to the limited number of data points. The Gaussian function is given in Equation (13).

(13) $$Y=Y_{0}+\alpha \times e^{-\frac{{\left(X-\beta \right)}^{2}}{{2\gamma }^{2}}}$$

where *Y* denotes RC, while α, β, and γ represent fitting parameters. The theoretical appropriate application rate of N fertilizer (*X* = β) was identified as the point at which the first derivative of the function equals zero. Because *X* = β represents a statistical estimate rather than a field-tested treatment, further validation is also required.

Before fitting, outliers were identified using the interquartile range method. Gaussian parameters (amplitude, mean, width, and offset) were estimated using the Levenberg–Marquardt nonlinear least-squares algorithm with 50 randomly perturbed initial values to improve convergence. The 95% confidence intervals were determined using asymptotic standard errors and bootstrap percentiles (1000 resamples). Model performance was evaluated based on residual normality (Shapiro–Wilk test), goodness of fit ($R^{2}\geq 0.90$), and leave-one-out cross-validation (LOOCV, $R_{pred}^{2}>0.80$).

### 2.7. Statistical Analysis

Residual normality was assessed using the Shapiro–Wilk test, and homogeneity of variances was verified using Levene’s test before one-way analysis of variance (ANOVA). Both assumptions were satisfied for all variables. When the one-way ANOVA was significant, treatment means were compared using Fisher’s least significant difference (LSD) tests at *p* < 0.05. Data on grain yield, yield components, quality traits, N uptake, and N-use efficiency of early and late rice were analyzed using ANOVA in IBM SPSS 26.0 (*p* < 0.05). The individual plot served as the experimental unit, with 18 plots per rice season. Early and late rice were analyzed separately owing to differences in growing conditions and rice varieties. Pearson correlation analysis was conducted to estimate the relationships among grain yields, yield components, nutrient accumulations, *PFP_N_*, and quality traits in the double-rice system.

Random forest modeling was performed to identify the key predictors of grain quality under the combined application of RSB and MV with reduced N inputs [32]. All analyses were conducted in R (version 4.3.3) using the “randomForest” package. Hyperparameter tuning and model validation were performed using the “caret” package. The dataset comprised eight predictors (yield components, nutrient accumulation, and partial factor productivity of nitrogen) with 18 observations per rice season (six treatments × three replicates). Model predictive stability was evaluated using LOOCV, repeated 10 times with different random seeds. The final R^2^ and root mean square error (RMSE) were calculated as the averages across the runs. Hyperparameter tuning focused on the number of variables randomly selected at each split (mtry = 2, 3, 4). The number of trees (ntree) was fixed at 500. Variable importance was quantified using the mean percentage increase in mean squared error (%IncMSE), and 95% confidence intervals were estimated through bootstrap resampling (1000 iterations). The optimal model was trained using all 18 observations and used for interpretation. Sigmaplot 14.0 and MS Excel 2016 were used to generate figures and tables, respectively.

## 3. Results

### 3.1. Rice Yield and Yield Components

**Figure 1:**
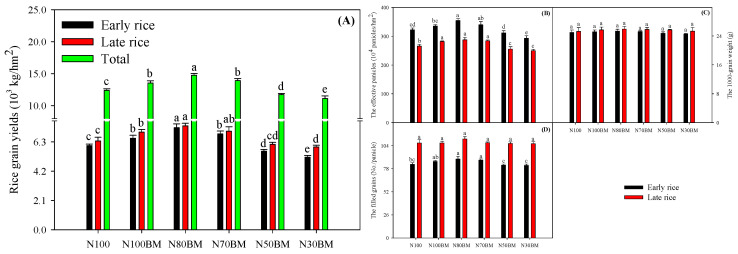
Effects of the combined application of RSB and MV with N reduction on grain yield and yield components of early and late rice. (**A**) Grain yield. (**B**) No. of effective panicles. (**C**) 1000-grain weight. (**D**) No. of filled grain per panicle. Results are expressed as mean ± SE (*n* = 3). Different letters indicate significant differences for the same indicator in the same rice season among different treatments (Fisher’s LSD, *p* < 0.05).

The combined application of RSB and MV with moderate N reduction before early-rice transplanting significantly increased rice yield in the double-cropping rice system (Figure 1A). Compared with the N_100_ treatment, the N_100_BM, N_80_BM, and N_70_BM treatments increased early-rice yield by 8.62%, 21.2%, and 13.8%, respectively, and late-rice yield by 9.88%, 16.9%, and 10.9%, respectively. Consequently, annual rice yield increased by 9.27%, 19.0%, and 12.3%. In contrast, the N_50_BM and N_30_BM treatments significantly reduced early-rice yield by 6.79% and 13.9%, respectively, and late-rice yield by 3.89% and 6.67%, respectively. Thus, annual rice yield decreased by 5.30% and 10.2% (*p* < 0.05).

Moreover, the combined application of RSB and MV with moderate N fertilization before early-rice transplanting significantly optimized rice yield components (Figure 1B–D). Compared with the N_100_ treatment, the N_80_BM and N_70_BM treatments increased the effective panicle number of early rice by 10.2% and 5.54%, respectively, and the number of filled grains per panicle by 7.11% and 5.78%, respectively. In late rice, the effective panicle number increased by 8.09% and 6.78%. In contrast, the N_30_BM treatment significantly reduced the effective panicle number of early and late rice by 9.08% and 6.02%, respectively, compared with the N_100_ treatment (*p* < 0.05).

### 3.2. N, P, and K Accumulation in Rice Grains

**Figure 2:**
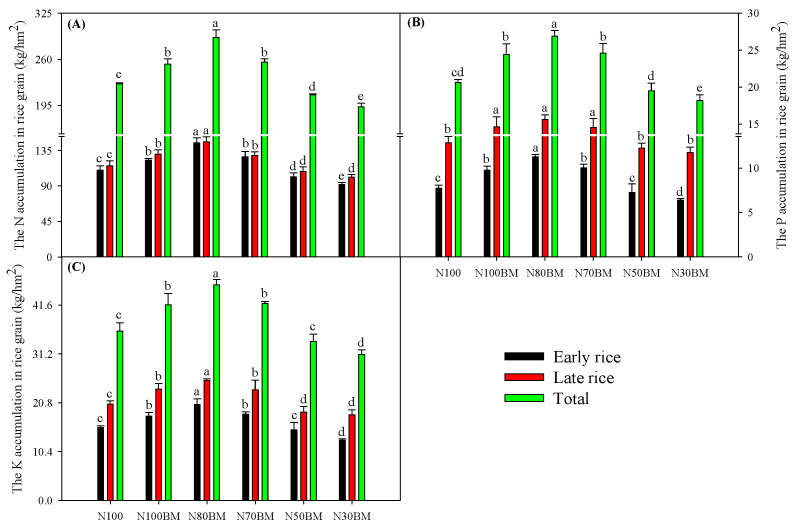
Effects of the combined application of RSB and MV with N reduction on the nutrient accumulations of early and late rice grain. (**A**) N accumulation in grain. (**B**) Phosphorus (P) accumulation in grain. (**C**) Potassium (K) accumulation in grain. Results are expressed as mean ± SE (*n* = 3). Different letters indicate significant differences for the same indicator in the same rice season among different treatments (Fisher’s LSD, *p* < 0.05).

The combined application of RSB and MV with an appropriate N reduction before early-rice transplanting substantially enhanced N, P, and K nutrients accumulation in both early- and late-rice grains (Figure 2). Compared with the N_100_ treatment, the N_100_BM, N_80_BM, and N_70_BM treatments (≤30% N reduction) increased N, P, and K accumulations in early-rice grains by 11.9–31.6%, 26.3–45.7%, and 15.7–31.5%, respectively. Similarly, N, P, and K accumulations in late-rice grains increased by 11.6–26.4%, 13.0–21.3%, and 14.9–24.5%, respectively. Consequently, the annual accumulations of these nutrients in rice grains increased by 12.4–20.7%, 18.5–30.4%, and 15.5–27.5%, respectively, with N_80_BM exhibiting the greatest increase among all treatments.

However, the N_50_BM and N_30_BM treatments reduced nutrient accumulation in early and late rice grain (Figure 2). Compared with the N_100_ treatment, the N_30_BM treatment substantially reduced N, P, and K accumulations in early-rice grains by 16.5%, 17.5%, and 17.4%, respectively, and in late-rice grains by 12.6%, 8.36%, and 11.1%, respectively. Consequently, annual N, P, and K accumulations in rice grains decreased by 14.5%, 11.8%, and 13.8%, respectively.

### 3.3. Partial Factor Productivity of N Fertilizer (PFP_N_)

**Figure 3:**
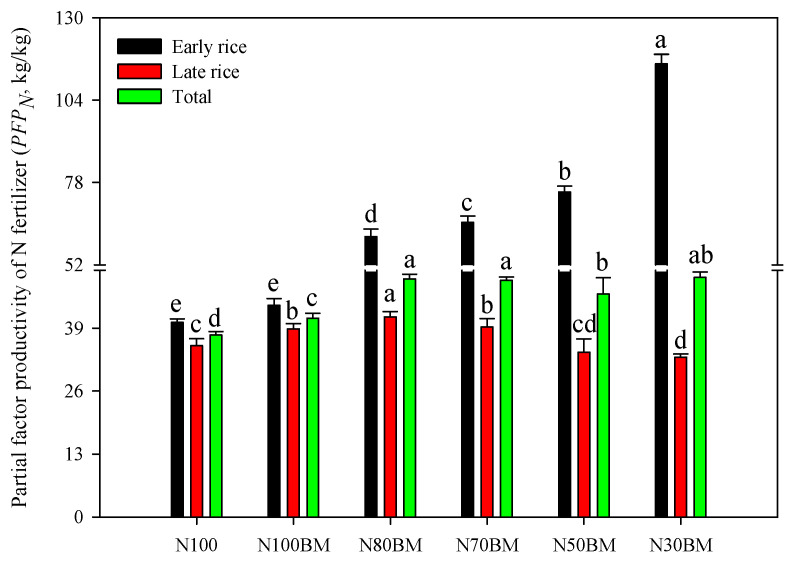
Effects of the combined application of RSB and MV with N reduction on partial factor productivity of N fertilizer (*PFP_N_*) of double rice grain. Results are expressed as mean ± SE (*n* = 3). Different letters indicate significant differences for the same indicator in the same rice season among different treatments (Fisher’s LSD, *p* < 0.05).

Compared with the N_100_ treatment, the combined application of RSB and MV under N-reduction rates before early-rice transplanting substantially increased *PFP_N_* in early rice by 8.62–187.6% (Figure 3). In late rice, the N_100_BM, N_80_BM, and N_70_BM treatments increased *PFP_N_* by 9.88–16.9%, leading to annual *PFP_N_* increases of 9.27–31.7%. In contrast, the N_30_BM treatment moderately reduced *PFP_N_* in late rice by 6.67% compared with the N_100_ treatment (*p* < 0.05).

### 3.4. Rice Grain Quality

#### 3.4.1. Grain Appearance Quality

The combined application of RSB and MV with appropriate N fertilization before early-rice transplanting substantially reduced the chalky grain rate and chalkiness degree of early rice (*p* < 0.05). However, no significant differences were observed in the length-to-width ratio of milled rice among treatments (*p* > 0.05; Table 2). Compared with the N_100_ treatment, the N_80_BM and N_70_BM treatments reduced the chalky grain rate by 27.3% and 9.34%, respectively, and the chalkiness degree by 25.9% and 6.61%, respectively. Conversely, the N_30_BM treatment increased the chalky grain rate and chalkiness degree by 7.43% and 13.3%, respectively.

This integrated practice had no significant effect on the length-to-width ratio of head rice in late rice (*p* > 0.05; Table 2). However, compared with the N_100_ treatment, a moderate N reduction (≤30%) greatly reduced the chalky grain rate and chalkiness degree of late rice by 11.4–18.8% and 15.6–25.9%, respectively. Notably, the N_80_BM treatment achieved the greatest reduction in both indicators. In contrast, the N_50_BM and N_30_BM treatments increased both the chalky grain rate and chalkiness degree of late rice. Specifically, the N_30_BM treatment increased the chalky grain rate and chalkiness degree by 6.17% and 15.1%, respectively (*p* < 0.05).

#### 3.4.2. Grain Milling Quality

**Figure 4:**
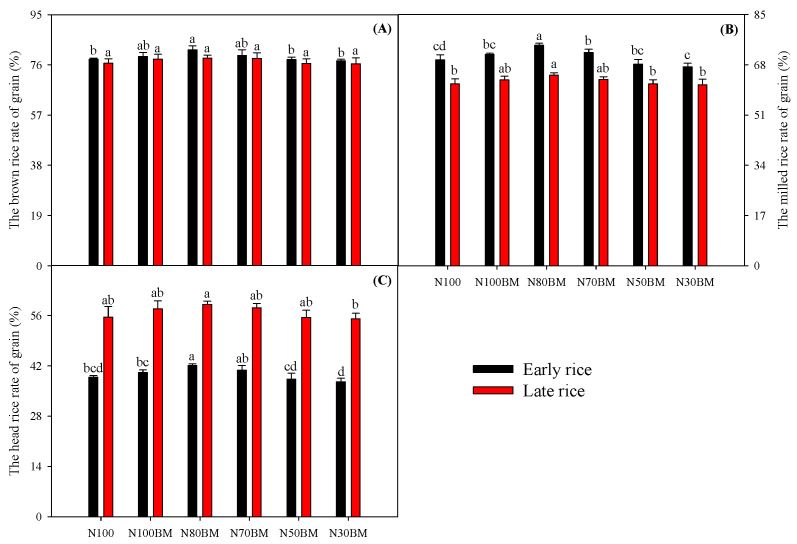
Effects of the combined application of RSB and MV with N reduction on grain milling quality of early rice and late rice. (**A**) Brown rice rate. (**B**) Milled rice rate. (**C**) Head rice rate. Results are expressed as mean ± SE (*n* = 3). Different letters indicate significant differences for the same indicator in the same rice season among different treatments (Fisher’s LSD, *p* < 0.05).

Compared with the N_100_ treatment, the N_70_BM and N_100_BM treatments increased the brown rice rate, milled rice rate, and head rice rate of both early and late rice. In contrast, the N_50_BM and N_30_BM treatments reduced these milling quality indicators (*p* > 0.05; Figure 4). Notably, the N_80_BM treatment moderately increased the milled rice rate and head rice rate of early rice by 7.16% and 8.29%, respectively, and the head rice rate of late rice by 6.37% compared with the N_100_ treatment (*p* < 0.05).

#### 3.4.3. Grain Cooking and Nutritional Quality

**Figure 5:**
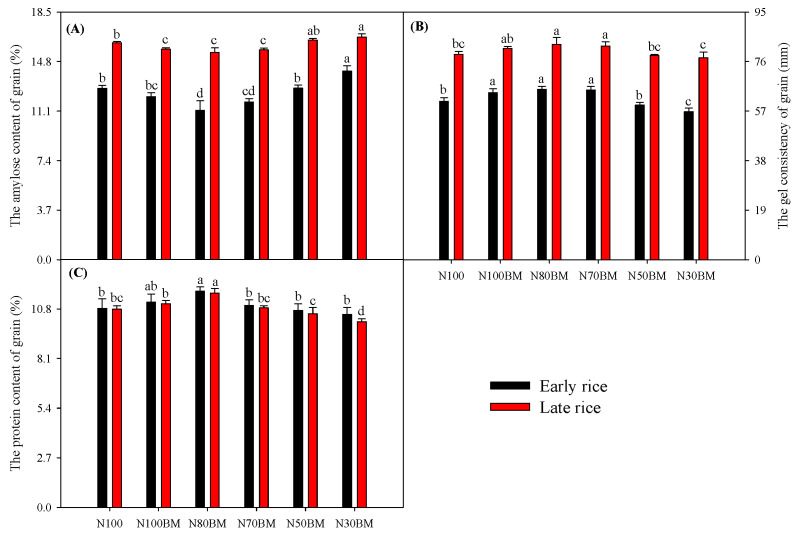
Effects of the combined application of RSB and MV with N reduction on the cooking and nutrition quality of early rice and late rice grain. (**A**) Amylose content. (**B**) Gel consistency. (**C**) Protein content. Results are expressed as mean ± SE (*n* = 3). Different letters indicate significant differences for the same indicator in the same rice season among different treatments (Fisher’s LSD, *p* < 0.05).

The combined application of RSB and MV with moderate N reduction before early-rice transplanting increased gel consistency and reduced amylose content in rice grains (Figure 5). Compared with the N_100_ treatment, the N_100_BM, N_80_BM, and N_70_BM treatments moderately increased gel consistency by 5.36%, 7.52%, and 7.18%, respectively. Moreover, these treatments reduced amylose content in early rice by 4.86%, 12.8%, and 7.97%. The N_50_BM treatment reduced gel consistency and protein content and increased amylose content in early rice (*p* > 0.05). In contrast, the N_30_BM treatment significantly reduced gel consistency by 6.54% and increased amylose content by 10.1% in early rice (*p* < 0.05). Compared with the N_100_ treatment, the N_80_BM treatment increased protein content by 8.61% and 8.14% in early and late rice, respectively.

Moreover, the trends in gel consistency, amylose content, and protein content of late rice were consistent with those of early rice, although no statistically significant differences were detected (*p* > 0.05; Figure 5).

### 3.5. Rapid Visco-Analyzer (RVA) Profile Traits of Rice Flour

**Figure 6:**
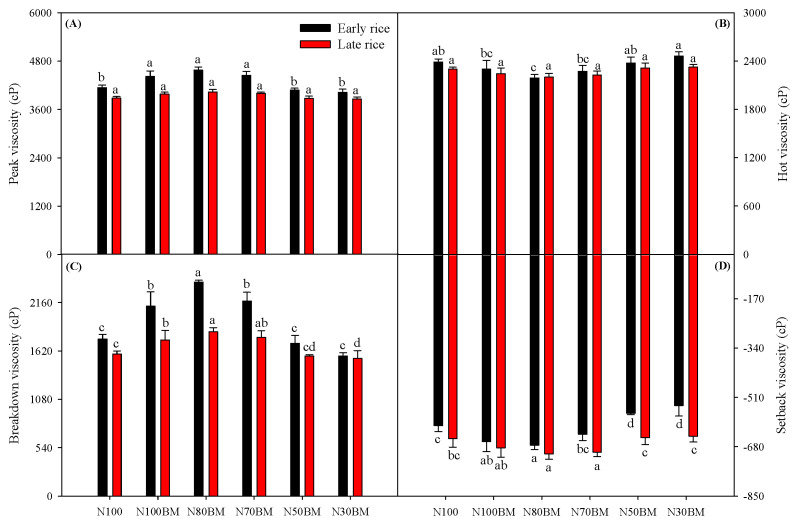
Effects of the combined application of RSB and MV with N reduction on the RVA profile traits of early rice and late rice flour. (**A**) Peak viscosity. (**B**) Hot viscosity. (**C**) Breakdown viscosity. (**D**) Setback viscosity. Results are expressed as mean ± SE (*n* = 3). Different letters indicate significant differences for the same indicator in the same rice season among different treatments (Fisher’s LSD, *p* < 0.05).

The combined application of RSB and MV with moderate N reduction improved the RVA profile of early-rice flour (Figure 6). Compared with the N_100_ treatment, the N_100_BM, N_80_BM, and N_70_BM treatments (70–100% N rates) increased the peak viscosity and breakdown value of early-rice flour by 6.85–10.6% and 21.0–36.1%, respectively. Additionally, these treatments reduced the hot viscosity and setback value by 3.54–8.26% and 4.89–11.2%, respectively. Among the treatments, N_80_BM exhibited the most favorable RVA profile. The N_50_BM and N_30_BM treatments had no significant effects on peak viscosity or hot viscosity (*p* > 0.05). However, the N_30_BM treatment reduced the breakdown value by 10.9% and increased the setback value by 11.3% in early-rice flour (*p* < 0.05).

Reduced N fertilization under combined RSB and MV application before early-rice transplanting had no significant effects on the peak viscosity or hot viscosity of late-rice flour (*p* > 0.05; Figure 6). However, compared with the N_100_ treatment, the N_100_BM, N_80_BM, and N_70_BM treatments (70–100% N rates) increased the breakdown value of late-rice flour by 9.70–15.5% and reduced the setback value by 4.91–8.18% (*p* < 0.05).

### 3.6. EW–TOPSIS-Based Multi-Objective Evaluation

The EW–TOPSIS model yielded highly consistent evaluation results for both early and late rice. The two-highest ranked treatments were N_80_BM and N_70_BM (Table 3). Higher evaluation scores indicated enhanced overall treatment performance. Specifically, under the selected EW-TOPSIS weighting structure, the N_80_BM was identified as the highest-ranked treatment among the tested treatments for early rice, achieving the highest comprehensive evaluation score (RC = 0.643). In contrast, the N_70_BM was identified as the highest-ranked treatment among the tested treatments for late rice (RC = 0.646). This result highlights that under the conditions of this study, a moderate N reduction (20–30%) combined with RSB and MV most effectively enhanced overall rice production performance among the tested treatments.

**Figure 7:**
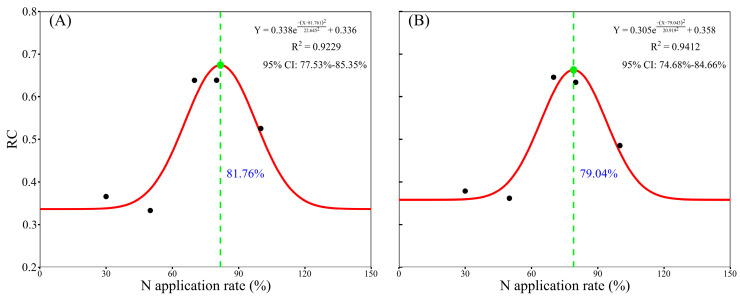
Relation between the relative closeness (RC) and application rates of N fertilizer of early rice (**A**) and late rice (**B**).

Furthermore, the relationship between N application rate and RC was well described by a Gaussian function (R^2^ = 0.9229 and 0.9412 for early rice and late rice, respectively; Figure 7). Under the tested conditions, the model predicted N application rates of 81.8% and 79.0% of the conventional N rate for early and late rice, which correspond to about 18.2% and 21.0% reduction, respectively. These values were close to the N application rate used in the tested N_80_BM treatment (80% of the conventional N rate) but were not evaluated as independent field treatments. Therefore, these predicted values should be regarded as model-based estimates requiring further experimental validation.

### 3.7. Pearson Correlation and Random Forest Analyses of Grain Yield, Nutrient Accumulation, PFP_N_, and Grain Quality

**Figure 8:**
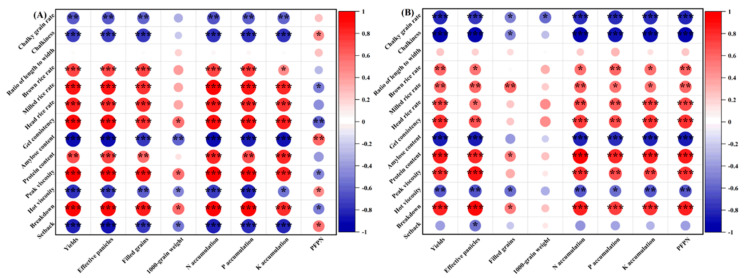
The Pearson correlation analysis among the grain yields, yields components, nutrients accumulations, *PFP_N_*, and quality of early rice (**A**) and late rice (**B**). * *p* < 0.05, ** *p* < 0.01, *** *p* < 0.001.

Grain yield, effective panicle number, filled grains per panicle, and nutrient accumulation (N, P, and K) in early rice were significantly and positively correlated with milling quality (brown rice rate, milled rice rate, and head rice rate), gel consistency, protein content, peak viscosity, and breakdown value. In contrast, these variables were strongly and negatively correlated with chalkiness degree, chalky grain rate, amylose content, hot viscosity, and setback value (*p* < 0.05 or *p* < 0.001). The 1000-grain weight was positively correlated with gel consistency, peak viscosity, and breakdown value (*p* < 0.05). Conversely, the 1000-grain weight was significantly and negatively correlated with amylose content, hot viscosity, and setback value (*p* < 0.05 or *p* < 0.01) (Figure 8A).

Moreover, grain yield, effective panicle number, N, P, and K accumulations, and *PFP_N_* in late rice were significantly positively correlated with milling quality (brown rice rate, milled rice rate, and head rice rate), gel consistency, protein content, peak viscosity, and breakdown value. In contrast, these variables were significantly negatively correlated with chalkiness degree, chalky grain rate, amylose content, and hot viscosity (*p* < 0.05 or *p* < 0.001). Filled grains per panicle were positively correlated with head rice rate, protein content, and breakdown value (*p* < 0.05; *p* < 0.01) but negatively correlated with chalkiness degree, chalky grain rate, and hot viscosity (*p* < 0.05; Figure 8B).

**Figure 9:**
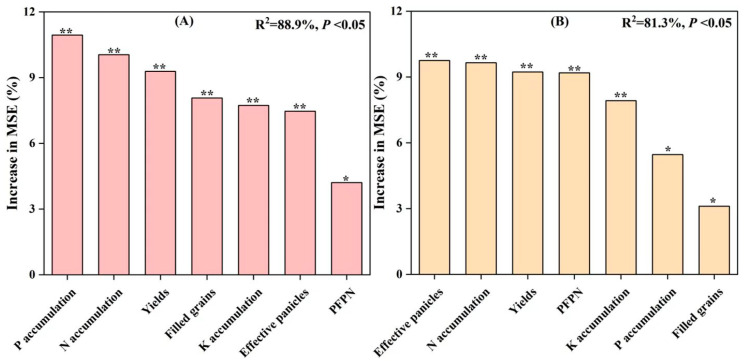
Random Forest regression modeling indicates the mean predictor importance of grain yield and nutrient performances affecting the grain quality of early rice (**A**) and late rice (**B**). MSE, mean square error. * *p* < 0.05, ** *p* < 0.01.

Additionally, the random forest model identified seven key predictors of grain quality in both early and late rice (Figure 9). The model accounted for 88.9% and 81.3% of the variation in grain quality in early and late rice, respectively. Nutrient accumulation (N, P, and K) emerged as the main predictor of grain quality, accounting for 28.7% and 23.0% of the total mean squared error (MSE). Grain yield and yield components (effective panicle number and filled grains per panicle) ranked second, contributing 24.8% and 22.1% of the total MSE, respectively. *PFP_N_* was the third-most vital predictor, accounting for 4.21% and 9.19% of the total MSE in early and late rice, respectively. However, because the random forest analysis was based on 18 observations per rice season, the predictor rankings should be interpreted as exploratory and should be confirmed using larger datasets.

## 4. Discussion

### 4.1. Rice Grain Yield and Its Components, Nutrient Accumulation, and PFP_N_

N is a key limiting factor for crop growth, development, and yield formation. Rational N management in paddy soils is crucial for achieving high yield, superior grain quality, and efficient nutrient utilization in rice [33]. Rice yield is mainly determined by effective panicle number, grains per panicle, and grain weight. Soil N availability strongly regulates tiller initiation and development [34]. However, both insufficient and excessive N supply can promote ineffective tillering, suppress effective tiller formation, and impair panicle development and floret differentiation [35]. Consequently, rice yield, grain quality, and NUE decrease. In this study, compared with conventional N fertilization (N_100_), moderate N reduction (≤30%) combined with the co-application of RSB and MV substantially increased N, P, and K accumulations in rice grains. This practice also increased effective panicle number and filled grains per panicle, thereby improving grain yield and *PFP_N_* in both early and late rice. The N_80_BM treatment showed the best overall performance among the tested treatments. In contrast, excessive N reduction (50–70%) significantly impaired overall agronomic performance (Figure 1, Figure 2 and Figure 3).

Previous studies have shown that combining high-C/N-ratio RSB with low-C/N-ratio MV exerts a synergistic C–N effect. This interaction stimulates decomposer enzyme activities, increases microbial abundance and diversity, accelerates MV decomposition during the early stages, and enhances soil N supply and retention capacity [21,36]. Under the integrated application of RSB and MV with sufficient N fertilization (≥70% of the conventional N rate), the fast-acting chemical N and slow-release organic N from MV may synergistically improve the synchrony between soil nutrient supply and plant demand. This synergistic effect may increase N, P, and K uptake and accumulation (Figure 2), thereby enhancing photosynthetic performance, canopy structure, and yield-related traits. Consequently, increased photosynthate production promotes effective tillering, floret differentiation, and assimilated translocation to grains [37], resulting in higher grain yield and NUE (Figure 1A and Figure 3). Conversely, low N input (30–50% of the conventional N rate) could intensify microbial competition for soil mineral N to meet stoichiometric demands, leading to increased N immobilization and reduced plant-available N [38]. The resulting N deficiency and nutrient supply–demand imbalance inhibited root growth and N uptake, leading to prolonged seedling recovery, delayed tillering, and increased floret abortion [35,39]. Consequently, N accumulation, effective panicle number, and filled grains per panicle decreased, resulting in lower grain yield (Figure 1 and Figure 2). However, because this study evaluated a combined management strategy, the individual effects of RSB, MV, and N reduction could not be isolated. Therefore, although the improvements in grain yield and nutrient uptake were robust, the underlying microbial and enzymatic mechanisms remain unclear and require further investigation. Future studies based on fully factorial experimental designs are needed to directly assess the individual and interactive effects of these management components.

Furthermore, grain yield is a key indicator of nutrient-use efficiency. In this study, a moderate N reduction, particularly under the N_80_BM treatment, achieved a favorable balance between grain yield and N productivity under the tested conditions (Figure 3). However, the increase in *PFP_N_* observed in early rice under N-reduced treatments was partly attributed to the lower N input. This was due to the substantially smaller reduction in grain yield than in the N application rate.

### 4.2. Rice Grain Quality and RVA Traits

Rational N fertilization plays a crucial role in regulating grain quality and RVA profile traits [25,40,41]. Grain appearance quality (such as the chalky grain rate and chalkiness degree) is a key determinant of market value. Milling quality (including brown rice rate, milled rice rate, and head-milled rice rate) largely depends on grain filling and endosperm development [42]. This study showed that compared with the N_100_ treatment, the N_70_BM, N80BM, and N_100_BM treatments substantially increased N, P, and K accumulation, improved milling quality, and reduced the chalky grain rate and chalkiness degree of rice (Table 2, Figure 2 and Figure 4). These improvements may be attributed to a balanced and stable nutrient supply, which enhanced photosynthetic performance, promoted endosperm development, and improved grain plumpness and uniformity [43]. These structural improvements increased grain compactness, thereby reducing grain breakage during subsequent milling operations [44]. Moreover, the results indicated that the number of filled grains per panicle was strongly positively correlated with milling quality (e.g., milled rice rate) but negatively correlated with appearance quality (e.g., chalky grain rate and chalkiness). Nutrient accumulation (N, P, and K) and yield components (particularly the filled grains per panicle) were identified as the main predictors of grain quality in both early and late rice (Figure 8 and Figure 9). Nevertheless, the causal relationships and underlying biological mechanisms require further investigation. Conversely, the N_50_BM and N_30_BM treatments were unfavorable for improving appearance quality and milling quality (Table 2 and Figure 4). This was likely attributed to insufficient N accumulation during the growing season, which accelerated leaf senescence and reduced photosynthate production.

Improved gel consistency and reduced amylose content contribute to enhanced rice cooking quality [45]. Gel consistency is negatively correlated with protein and amylose contents, while protein and amylose contents are positively correlated [46]. In this study, the co-application of RSB and MV with 70–100% N fertilizer reduced amylose content and increased gel consistency and protein content (Figure 5). This response may be attributed to changes in soil microbial diversity after RSB or MV incorporation and a more balanced nutrient supply [21]. These changes promoted nutrient uptake and utilization by rice plants and stimulated amino acid synthesis (e.g., lysine) in rice grains [47], thereby increasing grain protein content and reducing amylose content. However, these mechanisms were proposed based on previous studies and were not directly examined in the present study. Direct measurements of soil microbial community composition, enzyme activities, and nutrient transformation rates are needed to confirm these mechanistic interpretations. Moreover, the increase in gel consistency was likely attributed to the greater reduction in amylose content than the increase in protein content (Figure 5).

The rice RVA profile is a key indicator of cooking quality, and both cooking quality and RVA profile characteristics are largely determined by grain amylose and protein contents [48]. Amylose content is closely associated with the softness and stickiness of cooked rice. High protein content inhibits starch–water interactions and increases the initial gelatinization temperature [49]. Previous studies have shown that biochar application significantly increases rice peak viscosity and breakdown value [25]. In the present study, the co-application of RSB and MV with 70–100% N fertilizer increased peak viscosity and breakdown value but reduced hot viscosity and setback value (Figure 6). This indicates improved starch pasting properties. This response was likely attributed to the favorable growing conditions, improved crop performance, and enhanced panicle development under this management practice. In contrast, insufficient N supply under 30–50% N fertilization limited biomass accumulation, leading to lower peak viscosity and breakdown value but higher hot viscosity and setback value. These findings suggest that the effective tiller number and filled grains per panicle of early rice were positively correlated with peak viscosity and breakdown value but negatively correlated with hot viscosity and setback value. In late rice, the number of filled grains per panicle was positively correlated with grain protein content and breakdown value but negatively correlated with hot viscosity (Figure 8). Overall, the co-application of RSB and MV with an appropriate N fertilizer may coordinate the balance between protein and starch synthesis in rice grains.

### 4.3. Performances of Different Scenarios

Effective N management strategies in double-cropping rice systems should be evaluated based on multiple objectives, including grain yield, grain quality, and NUE. To achieve this, the EW–TOPSIS model was used [30]. Unlike subjective weighting methods, the EW approach objectively assigns indicator weights based on information entropy, thereby minimizing human bias and capturing the variability within the dataset. The EW–TOPSIS approach provides a transparent and reproducible framework for multi-criteria decision analysis. TOPSIS ranks alternatives according to their relative closeness to the positive and negative-ideal solutions. This anchoring effect enhances the robustness of the final ranking and reduces sensitivity to moderate fluctuations in indicator weights. Previous studies have shown that China could reduce national N consumption by 27% and maintain its rice yield target for 2030. Correspondingly, N inputs in early and late rice could be reduced by 17.7% and 26.7%, respectively [50]. In this study, 21 indicators (including yield, yield components, *PFP_N_*, and grain quality) were integrated into the EW–TOPSIS model for both early and late rice. The results revealed that the N_80_BM and N_70_BM treatments achieved the highest comprehensive scores for early rice and late rice, respectively (Table 3). However, the modest sample size (three replicates per treatment) limited the statistical power to detect small effect sizes. Additionally, simulation-based quantitative sensitivity analyses (e.g., global sensitivity analysis and weight perturbation tests) were not conducted to empirically assess the robustness of the rankings. Therefore, the results should be interpreted with appropriate caution.

Moreover, the Gaussian model-estimated N-reduction rates of 18.2% and 21.0% of the conventional N rate for early and late rice, respectively (Figure 7). These values were close to the N application rate used in the tested N_80_BM treatment. However, the estimated rates were not directly evaluated as field treatments. Furthermore, all main response variables were measured during a single double-rice cropping cycle in the fourth year of the experiment. Agroecosystem responses typically exhibit substantial interannual variability owing to fluctuations in soil quality, climate, weed pressure, pest abundance, and disease incidence. Therefore, the present results reflect responses under the specific conditions of this study and require further validation.

## 5. Conclusions

Under the fourth-year, single-site field conditions, RSB + MV combined with a moderate N reduction (≤30% of the conventional N rate) substantially improved grain yield, effective panicle number, nutrient accumulation (N, P, and K), and *PFP_N_* in both early and late rice. Additionally, this practice enhanced grain quality (appearance, milling, cooking, and nutritional quality) and RVA profile traits. Notably, the N_80_BM and treatment achieved the highest overall performance. In contrast, the N_50_BM and N_30_BM treatments exhibited limited effectiveness in improving agronomic performance and grain quality. The EW–TOPSIS analysis showed that the N_80_BM and N_70_BM treatments achieved the highest comprehensive scores for early and late rice. The Gaussian model estimated N reductions of 18.2% and 21.0% under RSB + MV for early and late rice, respectively. These model-based estimates were close to the N application rate used in the tested N_80_BM treatment but required further field validation. Grain yield, yield components, and nutrient accumulation were identified as the primary predictors of grain quality in both early and late rice.

These results were derived from a single double-rice cropping cycle in the fourth year of the field experiment after the treatment-establishment period. Hence, multi-site, long-term (≥10 years) field experiments are still needed to verify the validity, interannual stability, and generalizability of the observed response patterns. Additionally, the proposed mechanisms underlying C–N synergy remain unclear and require direct validation through targeted measurements of soil microbial communities, enzyme activities, and nutrient transformation processes.

## Figures and Tables

**Table 1:** Basic properties of RSB and MV.

Materials	pH (1:2.5)	Total C (g/kg)	Total N (g/kg)	C/N Ratio	Specific Area (m^2^/g)
RSB	9.69	758.4	12.3	61.7	41.3
MV	/	423.2	29.1	14.5	/

/ means no data.

**Table 2:** Effects of the combined application of RSB and MV with N reduction on the appearance quality of early- and late-rice grains.

Treatments	Chalky Grain Rate (%)		Chalkiness (%)		Ratio of Length to Width	
	Early Rice	Late Rice	Early Rice	Late Rice	Early Rice	Late Rice
N_100_	11.5 ± 0.24 b	8.49 ± 0.60 a	3.35 ± 0.18 b	2.45 ± 0.16 c	3.04 ± 0.05 a	3.67 ± 0.05 a
N_100_BM	11.4 ± 0.28 b	7.52 ± 0.20 b	3.22 ± 0.36 bc	2.07 ± 0.11 d	3.09 ± 0.02 a	3.68 ± 0.05 a
N_80_BM	8.37 ± 0.62 d	6.90 ± 0.59 c	2.49 ± 0.23 d	1.82 ± 0.12 e	3.09 ± 0.04 a	3.71 ± 0.04 a
N_70_BM	10.4 ± 0.69 c	7.28 ± 0.68 b	3.13 ± 0.30 c	1.97 ± 0.12 d	3.09 ± 0.02 a	3.71 ± 0.02 a
N_50_BM	11.7± 0.90 ab	8.66 ± 0.37 a	3.41 ± 0.19 b	2.60 ± 0.15 b	3.11 ± 0.02 a	3.71 ± 0.02 a
N_30_BM	12.4 ± 0.23 a	8.83 ± 0.92 a	3.80 ± 0.21 a	2.82 ± 0.12 a	3.08 ± 0.02 a	3.68 ± 0.05 a

Results are expressed as mean ± SE (*n* = 3). Different letters indicate significant differences for the same indicator in the same rice season among different treatments (Fisher’s LSD, *p* < 0.05).

**Table 3:** The Euclidean distances ${ED}_{i}^{+}$ and ${ED}_{i}^{-}$ between each scheme value and the ideal point and the relative closeness ${RC}_{i}$ of each target based on EW-TOPSIS model.

Treatments	Early Rice				Late Rice			
	${ED}_{i}^{+}$	${ED}_{i}^{-}$	${RC}_{i}$	*Rank*	${ED}_{i}^{+}$	${ED}_{i}^{-}$	${RC}_{i}$	*Rank*
N_100_	0.087	0.045	0.344	5	0.072	0.054	0.431	4
N_100_BM	0.058	0.076	0.566	3	0.068	0.063	0.485	3
N_80_BM	0.055	0.097	0.643	1	0.053	0.091	0.633	2
N_70_BM	0.048	0.083	0.631	2	0.046	0.081	0.646	1
N_50_BM	0.094	0.041	0.304	6	0.084	0.047	0.362	6
N_30_BM	0.098	0.055	0.353	4	0.083	0.052	0.379	5

## Data Availability

The original contributions presented in this study are included in the article/Supplementary Material. Further inquiries can be directed to the corresponding author.

## Supplementary Materials

The following supporting information can be downloaded at: https://www.mdpi.com/article/10.3390/foods15132354/s1. Table S1. Indicators system for entropy-weighted TOPSIS evaluation of multi-objective evaluation in double-rice cropping system. Table S2. The normalized matrix used to create the final ranking for early rice. Table S3. The weighted normalized matrix used to create the final ranking for early rice. Table S4. The normalized matrix used to create the final ranking of late rice. Table S5. The weighted normalized matrix used to create the final ranking of late rice. Table S6. Random forest input data of early rice (ER). Table S7. Random forest input data of late rice (LR). Table S8. Gaussian fitting data. Figure S1. The field location experimental site. Figure S2. Experimental design schematic.
