# Supplementary material for: Evaluating Nitrogen Reduction Under Combined Rice Straw Biochar and Milk Vetch Application: A Multi-Objective Assessment of Rice Yield, Grain Quality, and Partial Factor Productivity of Nitrogen Fertilizer in a Double-Rice Cropping System
Source: Foods. 2026 Jul 2;15(13):2354. doi: 10.3390/foods15132354 (PMC13360624; doi:10.3390/foods15132354)
Supplement: Supplementary file 1 [file foods-15-02354-s001.zip › EW-TOPSIS indicator matrix, entropy weights, normalized values.pdf]

Table S1. Indicators system for entropy-weighted TOPSIS evaluation of multi-objective evaluation in double-rice cropping system.

Indicator	Unit	Direction	Entropy weights	
			Early rice	Late rice
Grain yield	kg/hm^2	Positive	0.011	0.014
Effective panicles	10^4 panicles/hm^2	Positive	0.048	0.048
Filled grains	No./panicle	Positive	0.090	0.092
1000-grain weight	g	Positive	0.128	0.126
N accumulation	kg/hm^2	Positive	0.013	0.012
P accumulation	kg/hm^2	Positive	0.013	0.015
K accumulation	kg/hm^2	Positive	0.012	0.016
PFP_N	kg/kg	Positive	0.067	0.058
Chalky grain rate	%	Negative	0.026	0.024
Chalkiness	%	Negative	0.027	0.027
Ratio of length to width	/	Positive	0.015	0.020
Brown rice rate	%	Positive	0.022	0.022
Milled rice rate	%	Positive	0.014	0.021
Head rice rate	%	Positive	0.017	0.015
Gel consistency	mm	Positive	0.011	0.013
Protein content	%	Negative	0.141	0.142
Amylose content	%	Negative	0.022	0.028
Peak viscosity	cP	Positive	0.053	0.051
Hot viscosity	cP	Negative	0.102	0.097
Breakdown	cP	Positive	0.124	0.122
Setback	cP	Negative	0.163	0.158

Table S2. The normalized matrix used to create the final ranking for early rice.

Treat	Yield	EP	FG	TW	NA	PA	KA	PFP_N	CGR	Ch	R	BR	MR	HR	GC	PC	AC	PV	HV	BV	SV
	0.32	0.35	0.47	0.78	0.16	0.18	0.25	0.00	0.89	0.18	0.16	0.42	0.28	0.49	0.46	1.00	0.33	0.12	0.55	0.26	0.40
N_{100}	0.45	0.43	0.09	0.00	0.44	0.31	0.42	0.00	0.38	0.53	0.00	0.19	0.50	0.48	0.52	0.46	0.41	0.14	0.45	0.23	0.01
	0.43	0.60	0.30	0.44	0.46	0.35	0.43	0.00	0.00	0.21	0.00	0.06	0.32	0.00	0.31	0.37	0.63	0.36	0.00	0.22	0.65
	0.45	0.43	1.00	0.24	0.49	0.55	1.00	0.03	0.28	0.92	0.00	0.09	0.66	0.83	0.74	0.19	0.57	0.76	0.42	0.64	0.91
N_{100}BM	0.80	0.99	0.42	0.87	0.68	0.76	0.66	0.06	0.38	0.41	0.54	0.50	0.48	0.61	1.00	0.52	0.64	0.83	0.40	0.71	0.55
	0.71	0.66	0.55	1.00	0.61	0.79	0.24	0.05	0.21	0.16	0.86	0.68	0.65	0.30	0.58	0.56	0.79	0.74	0.88	0.78	0.71
	1.00	1.00	0.85	1.00	1.00	1.00	0.98	0.30	1.00	1.00	0.88	1.00	1.00	1.00	1.00	0.00	1.00	1.00	0.95	1.00	1.00
N_{80}BM	1.00	1.00	1.00	0.96	1.00	1.00	1.00	0.26	1.00	1.00	1.00	1.00	1.00	1.00	0.91	0.00	1.00	1.00	1.00	1.00	0.88
	1.00	1.00	0.85	0.38	1.00	1.00	1.00	0.26	1.00	1.00	0.46	1.00	1.00	1.00	0.83	0.00	1.00	1.00	1.00	1.00	1.00
	0.59	0.61	0.98	0.45	0.43	0.68	0.57	0.30	0.61	0.91	0.07	0.00	0.57	0.54	0.77	0.74	0.66	0.66	1.00	0.79	0.71
N_{70}BM	0.94	0.94	0.57	1.00	0.81	0.69	0.92	0.36	0.33	0.36	0.70	0.73	0.74	1.00	0.86	0.45	0.81	0.82	0.77	0.80	1.00
	0.87	0.72	1.00	0.77	0.79	0.88	0.76	0.34	0.68	0.43	0.84	0.63	0.76	0.70	1.00	0.34	0.93	0.89	0.31	0.69	0.57
	0.18	0.04	0.00	0.55	0.09	0.21	0.30	0.46	0.16	0.15	1.00	0.08	0.00	0.00	0.11	0.85	0.34	0.00	0.45	0.14	0.00
N_{50}BM	0.21	0.35	0.25	0.10	0.12	0.33	0.07	0.46	0.25	0.51	0.48	0.35	0.10	0.59	0.25	1.00	0.40	0.09	0.31	0.15	0.07
	0.22	0.48	0.02	0.00	0.34	0.00	0.50	0.46	0.44	0.13	1.00	0.00	0.43	0.09	0.40	0.26	0.61	0.22	0.10	0.17	0.36
	0.00	0.00	0.33	0.00	0.00	0.00	0.00	1.00	0.00	0.00	0.10	0.25	0.18	0.12	0.00	0.72	0.00	0.04	0.00	0.00	0.12
N_{30}BM	0.00	0.00	0.00	0.40	0.00	0.00	0.00	1.00	0.00	0.00	0.85	0.00	0.00	0.00	0.00	0.87	0.00	0.00	0.00	0.00	0.00
	0.00	0.00	0.00	0.09	0.00	0.00	0.00	1.00	0.28	0.00	0.60	0.09	0.00	0.10	0.00	1.00	0.00	0.00	0.04	0.00	0.00

Note: EP, effective panicle number; FG, filled grains per panicle; TW, 1000-grain weight; NA, N accumulation; PA, P accumulation; KA, K accumulation; PFP_N, partial factor productivity of N fertilizer; CGR, chalky grain rate; Ch, chalkiness; R, ratio of length to width; BR, brown rice rate; MR, milled rice rate; HR, head rice rate; GC, gel consistency; PC, protein content; AC, amylose content; PV, peak viscosity; HV, hot viscosity; BV, breakdown value; SV, setback value.

Table S3. The weighted normalized matrix used to create the final ranking for early rice.

Treat	yield	EP	FG	TW	NA	PA	KA	PFPN	CGR	Ch	R	BR	MR	HR	GC	PC	AC	PV	HV	BV	SV
	0.001	0.007	0.011	0.030	0.001	0.001	0.001	0.000	0.008	0.002	0.002	0.007	0.002	0.003	0.002	0.039	0.003	0.003	0.015	0.011	0.020
N_{100}	0.001	0.006	0.003	0.000	0.002	0.001	0.002	0.000	0.004	0.004	0.000	0.001	0.002	0.001	0.002	0.018	0.003	0.003	0.015	0.010	0.000
	0.001	0.008	0.010	0.021	0.001	0.002	0.002	0.000	0.000	0.003	0.000	0.001	0.001	0.000	0.001	0.023	0.003	0.005	0.000	0.010	0.031
	0.002	0.009	0.024	0.009	0.004	0.003	0.004	0.001	0.002	0.008	0.000	0.001	0.004	0.005	0.003	0.007	0.004	0.016	0.012	0.027	0.046
N_{100}BM	0.003	0.013	0.016	0.036	0.003	0.003	0.003	0.002	0.004	0.003	0.001	0.002	0.002	0.001	0.003	0.020	0.005	0.016	0.014	0.031	0.038
	0.002	0.008	0.018	0.048	0.002	0.004	0.001	0.002	0.002	0.003	0.002	0.006	0.002	0.003	0.002	0.035	0.004	0.011	0.042	0.034	0.034
	0.005	0.021	0.020	0.039	0.007	0.005	0.004	0.009	0.009	0.009	0.012	0.016	0.007	0.006	0.004	0.000	0.008	0.022	0.026	0.042	0.050
N_{80}BM	0.003	0.013	0.039	0.040	0.004	0.003	0.005	0.008	0.011	0.008	0.002	0.004	0.005	0.002	0.003	0.000	0.007	0.019	0.034	0.044	0.061
	0.003	0.013	0.028	0.018	0.003	0.006	0.004	0.008	0.010	0.016	0.001	0.009	0.003	0.011	0.003	0.000	0.005	0.015	0.048	0.043	0.048
	0.003	0.013	0.024	0.017	0.003	0.003	0.002	0.009	0.005	0.008	0.001	0.000	0.004	0.003	0.003	0.028	0.005	0.014	0.027	0.033	0.036
N_{70}BM	0.003	0.012	0.023	0.041	0.003	0.002	0.004	0.012	0.004	0.003	0.002	0.003	0.003	0.002	0.003	0.018	0.006	0.016	0.026	0.036	0.070
	0.003	0.009	0.034	0.037	0.002	0.005	0.003	0.011	0.007	0.007	0.002	0.006	0.002	0.008	0.003	0.021	0.005	0.013	0.015	0.030	0.027
	0.001	0.001	0.000	0.021	0.001	0.001	0.001	0.014	0.001	0.001	0.013	0.001	0.000	0.000	0.000	0.033	0.003	0.000	0.012	0.006	0.000
N_{50}BM	0.001	0.005	0.010	0.004	0.001	0.001	0.000	0.015	0.003	0.004	0.001	0.002	0.000	0.001	0.001	0.039	0.003	0.002	0.011	0.007	0.005
	0.001	0.006	0.001	0.000	0.001	0.000	0.002	0.015	0.004	0.002	0.002	0.000	0.001	0.001	0.001	0.016	0.003	0.003	0.005	0.007	0.017
	0.000	0.000	0.008	0.000	0.000	0.000	0.000	0.031	0.000	0.000	0.001	0.004	0.001	0.001	0.000	0.028	0.000	0.001	0.000	0.000	0.006
N_{30}BM	0.000	0.000	0.000	0.017	0.000	0.000	0.000	0.032	0.000	0.000	0.002	0.000	0.000	0.000	0.000	0.034	0.000	0.000	0.000	0.000	0.000
	0.000	0.000	0.000	0.004	0.000	0.000	0.000	0.032	0.003	0.000	0.001	0.001	0.000	0.001	0.000	0.063	0.000	0.000	0.002	0.000	0.000

Note: EP, effective panicle number; FG, filled grains per panicle; TW, 1000-grain weight; NA, N accumulation; PA, P accumulation; KA, K accumulation; PFP_N, partial factor productivity of N fertilizer; CGR, chalky grain rate; Ch, chalkiness; R, ratio of length to width; BR, brown rice rate; MR, milled rice rate; HR, head rice rate; GC, gel consistency; PC, protein content; AC, amylose content; PV, peak viscosity; HV, hot viscosity; BV, breakdown value; SV, setback value.

Table S4. The normalized matrix used to create the final ranking of late rice.

Treat	yield	EP	FG	TW	NA	PA	KA	PFPN	CGR	Ch	R	BR	MR	HR	GC	PC	AC	PV	HV	BV	SV
	0.46	0.38	0.07	0.00	0.43	0.49	0.55	0.46	0.89	0.18	0.16	0.42	0.28	0.49	0.46	1.00	0.33	0.12	0.55	0.26	0.40
N_{100}	0.13	0.67	0.67	0.16	0.21	0.11	0.00	0.13	0.32	0.37	0.89	0.00	0.18	0.00	0.02	0.60	0.30	0.67	0.47	0.46	0.91
	0.27	0.36	0.00	0.47	0.32	0.31	0.53	0.27	0.63	0.14	0.00	0.55	0.42	0.41	0.38	0.55	0.14	0.24	0.27	0.18	0.70
	0.58	0.68	0.59	0.27	0.52	0.70	0.58	0.58	0.28	0.92	0.00	0.09	0.66	0.83	0.74	0.19	0.57	0.76	0.42	0.64	0.91
N_{100}BM	0.74	1.00	0.00	0.00	0.72	0.98	0.76	0.74	0.59	0.78	0.94	0.72	0.00	0.65	0.47	0.27	0.90	1.00	0.39	0.61	1.00
	0.78	0.89	0.87	1.00	0.74	0.59	0.91	0.78	0.90	0.78	0.18	0.47	0.37	1.00	0.68	0.30	0.52	0.83	0.55	0.79	0.22
	1.00	1.00	1.00	0.37	1.00	0.99	1.00	1.00	1.00	1.00	0.88	1.00	1.00	1.00	1.00	0.00	1.00	1.00	0.95	1.00	1.00
N_{80}BM	1.00	0.94	1.00	1.00	1.00	1.00	1.00	1.00	1.00	1.00	1.00	0.61	1.00	1.00	1.00	0.00	1.00	0.86	1.00	1.00	0.32
	1.00	1.00	1.00	0.49	1.00	1.00	1.00	1.00	1.00	1.00	1.00	1.00	1.00	0.93	1.00	0.00	1.00	1.00	0.70	1.00	0.86
	0.87	0.80	0.62	1.00	0.63	1.00	0.83	0.87	0.61	0.91	0.07	0.00	0.57	0.54	0.77	0.74	0.66	0.66	1.00	0.79	0.71
N_{70}BM	0.41	0.92	0.15	0.14	0.44	0.31	0.54	0.41	0.58	0.81	0.56	0.32	0.11	0.78	0.68	0.47	0.90	0.92	0.91	0.97	0.91
	0.98	1.00	0.53	0.73	0.81	0.85	0.90	0.98	0.80	0.78	0.75	0.94	0.65	0.68	0.89	0.41	0.48	0.75	1.00	0.89	1.00
	0.12	0.26	0.04	0.59	0.13	0.00	0.02	0.12	0.16	0.15	1.00	0.08	0.00	0.00	0.11	0.85	0.34	0.00	0.45	0.14	0.00
N_{50}BM	0.13	0.33	0.17	0.26	0.23	0.10	0.05	0.13	0.00	0.35	0.59	0.41	0.69	0.86	0.23	0.53	0.00	0.75	0.00	0.14	0.72
	0.10	0.00	0.87	0.63	0.13	0.38	0.25	0.10	0.38	0.00	0.78	0.06	0.00	0.28	0.28	0.80	0.05	0.00	0.37	0.00	0.00
	0.00	0.00	0.00	0.48	0.00	0.12	0.00	0.00	0.00	0.00	0.10	0.25	0.18	0.12	0.00	0.72	0.00	0.04	0.00	0.00	0.12
N_{30}BM	0.00	0.00	0.32	0.61	0.00	0.00	0.08	0.00	0.16	0.00	0.00	1.00	0.70	0.78	0.00	1.00	0.00	0.00	0.44	0.00	0.00
	0.00	0.24	0.49	0.00	0.00	0.00	0.00	0.00	0.00	0.06	0.56	0.00	0.03	0.00	0.00	1.00	0.00	0.34	0.00	0.16	0.22

Note: EP, effective panicle number; FG, filled grains per panicle; TW, 1000-grain weight; NA, N accumulation; PA, P accumulation; KA, K accumulation; PFP_N, partial factor productivity of N fertilizer; CGR, chalky grain rate; Ch, chalkiness; R, ratio of length to width; BR, brown rice rate; MR, milled rice rate; HR, head rice rate; GC, gel consistency; PC, protein content; AC, amylose content; PV, peak viscosity; HV, hot viscosity; BV, breakdown value; SV, setback value.

Table S5. The weighted normalized matrix used to create the final ranking of late rice.

Treat	yield	EP	FG	TW	NA	PA	KA	PFPN	CGR	Ch	R	BR	MR	HR	GC	PC	AC	PV	HV	BV	SV
	0.002	0.006	0.003	0.000	0.002	0.002	0.003	0.009	0.008	0.002	0.002	0.007	0.002	0.003	0.002	0.039	0.003	0.003	0.015	0.011	0.020
N_{100}	0.001	0.007	0.027	0.010	0.001	0.001	0.000	0.003	0.003	0.002	0.004	0.000	0.002	0.000	0.000	0.031	0.003	0.007	0.015	0.017	0.035
	0.001	0.006	0.000	0.017	0.001	0.001	0.001	0.005	0.004	0.002	0.000	0.003	0.003	0.001	0.001	0.025	0.002	0.004	0.009	0.008	0.039
	0.002	0.010	0.023	0.013	0.002	0.003	0.003	0.011	0.002	0.008	0.000	0.001	0.004	0.005	0.003	0.007	0.004	0.016	0.012	0.027	0.046
N_{100}BM	0.005	0.011	0.000	0.000	0.003	0.007	0.007	0.017	0.005	0.005	0.004	0.005	0.000	0.003	0.003	0.014	0.009	0.010	0.012	0.022	0.038
	0.004	0.014	0.021	0.036	0.003	0.002	0.003	0.015	0.005	0.009	0.001	0.002	0.003	0.003	0.002	0.014	0.008	0.015	0.018	0.034	0.012
	0.004	0.014	0.039	0.017	0.004	0.005	0.005	0.019	0.009	0.009	0.012	0.016	0.007	0.006	0.004	0.000	0.008	0.022	0.026	0.042	0.050
N_{80}BM	0.006	0.011	0.041	0.061	0.005	0.007	0.009	0.023	0.009	0.007	0.005	0.004	0.010	0.004	0.007	0.000	0.010	0.008	0.031	0.036	0.012
	0.005	0.016	0.025	0.018	0.004	0.003	0.003	0.019	0.006	0.011	0.003	0.005	0.007	0.003	0.003	0.000	0.015	0.018	0.024	0.044	0.048
	0.003	0.012	0.024	0.047	0.003	0.005	0.004	0.016	0.005	0.008	0.001	0.000	0.004	0.003	0.003	0.028	0.005	0.014	0.027	0.033	0.036
N_{70}BM	0.003	0.010	0.006	0.009	0.002	0.002	0.005	0.010	0.005	0.005	0.003	0.002	0.001	0.003	0.005	0.024	0.009	0.009	0.029	0.035	0.035
	0.005	0.016	0.013	0.026	0.004	0.003	0.003	0.019	0.005	0.009	0.002	0.004	0.005	0.002	0.003	0.019	0.007	0.013	0.034	0.039	0.056
	0.000	0.004	0.002	0.027	0.001	0.000	0.000	0.002	0.001	0.001	0.013	0.001	0.000	0.000	0.000	0.033	0.003	0.000	0.012	0.006	0.000
N_{50}BM	0.001	0.004	0.007	0.016	0.001	0.001	0.000	0.003	0.000	0.002	0.003	0.003	0.007	0.004	0.002	0.028	0.000	0.007	0.000	0.005	0.027
	0.000	0.000	0.021	0.023	0.001	0.001	0.001	0.002	0.002	0.000	0.003	0.000	0.000	0.001	0.001	0.037	0.001	0.000	0.012	0.000	0.000
	0.000	0.000	0.000	0.022	0.000	0.001	0.000	0.000	0.000	0.000	0.001	0.004	0.001	0.001	0.000	0.028	0.000	0.001	0.000	0.000	0.006
N_{30}BM	0.000	0.000	0.013	0.037	0.000	0.000	0.001	0.000	0.001	0.000	0.000	0.007	0.007	0.003	0.000	0.052	0.000	0.000	0.014	0.000	0.000
	0.000	0.004	0.012	0.000	0.000	0.000	0.000	0.000	0.000	0.001	0.002	0.000	0.000	0.000	0.000	0.046	0.000	0.006	0.000	0.007	0.012

Note: EP, effective panicle number; FG, filled grains per panicle; TW, 1000-grain weight; NA, N accumulation; PA, P accumulation; KA, K accumulation; PFP_N, partial factor productivity of N fertilizer; CGR, chalky grain rate; Ch, chalkiness; R, ratio of length to width; BR, brown rice rate; MR, milled rice rate; HR, head rice rate; GC, gel consistency; PC, protein content; AC, amylose content; PV, peak viscosity; HV, hot viscosity; BV, breakdown value; SV, setback value.
