# Supplementary figures and images for: Evaluating Nitrogen Reduction Under Combined Rice Straw Biochar and Milk Vetch Application: A Multi-Objective Assessment of Rice Yield, Grain Quality, and Partial Factor Productivity of Nitrogen Fertilizer in a Double-Rice Cropping System
Source: Foods. 2026 Jul 2;15(13):2354. doi: 10.3390/foods15132354 (PMC13360624; doi:10.3390/foods15132354)

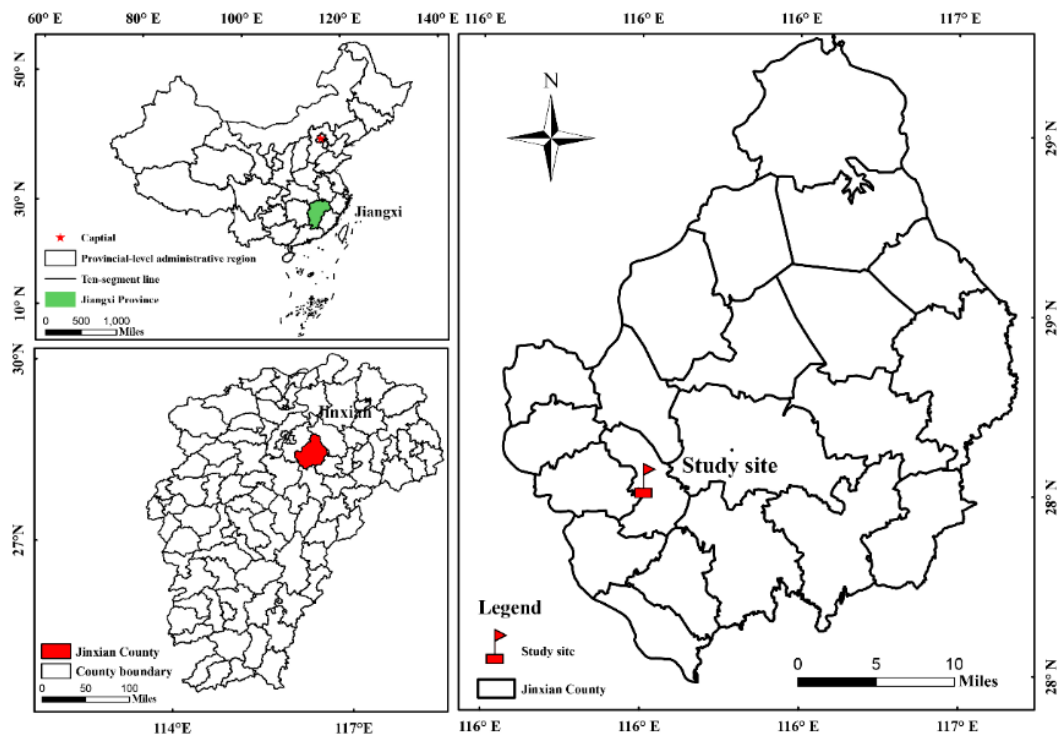

Figure. S1 The field location experimental site

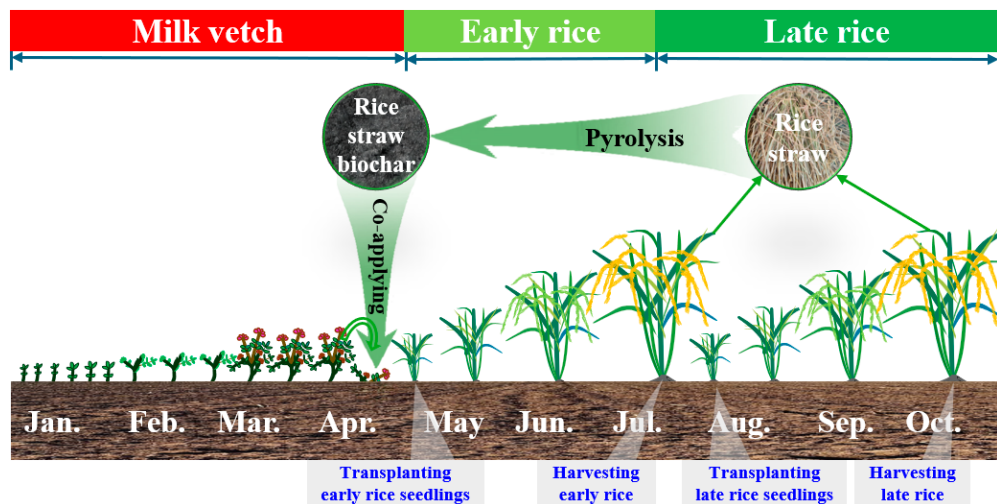

Figure. S2 Experimental design schematic

Supplement: Supplementary file 1 [file foods-15-02354-s001.zip › Supplemental figures S1 and S2.pdf]
